# Supplementary material for: Controlling the Surface Morphology of Two-Dimensional Nano-Materials upon Molecule-Mediated Crystal Growth
Source: Nanomaterials (Basel). 2023 Aug 18;13(16):2363. doi: 10.3390/nano13162363 (PMC10458610; doi:10.3390/nano13162363)
Supplement: Supplementary file 1 [file nanomaterials-13-02363-s001.zip › nanomaterials-2502729-supplementary.pdf]

Supporting Information for:

Controlling the Surface Morphology of Two Dimensional Nanomaterials upon Molecule-Mediated Crystal Growth

Tetsuo Yamaguchi,[1] Hyoung-Jun Kim,[2] Hee Jung Park,[3] Taeho Kim,[1] Zubair Khalid,[1] Jin Kuen Park,[4]* Jae-Min Oh[1]*

[1]*Department of Energy and Materials Engineering, Dongguk University-Seoul, Seoul 04620, Korea*

E-mail: *jaemin.oh@dongguk.edu*

[2]*Research Institute, National Cancer Center, 323, Ilsan-ro, Goyang, Gyeonggi 10408, Republic of Korea*

[3] *KBSI Western Seoul Center, University-Industry Cooperation Building, 150 Bugahyeon-ro, Seodaemun-gu, Seoul 03759, Republic of Korea*

[4] *Department of Chemistry, Hankuk University of Foreign Studies, Yongin, 17035, Gyeonggi-do, Republic of Korea.*

E-mail: *jinkpark@hufs.ac.kr*

1. Experimental

2. AFM images

1. Experimental

Table S1. Changes of pH values of methyl orange (MO) aqueous suspensions during the reconstruction of the LDHs.

Concentration (eq)	0.2	0.5	3.0
0 h (before adding LDO)	8.47	8.48	7.93
0.5 h	11.10	10.65	11.92
4 h	11.51	11.61	11.96
12 h	11.56	11.75	11.87

Table S2. Particle sizes (nm) of the reconstructed LDH_{2000} in CO_3^{2-} estimated by Sherrer's equation.

	0.2 eq	3 eq
0.5 h	5.40 nm	13.5 nm
4 h	4.66 nm	19.3 nm
12 h	8.63 nm	9.01 nm

Table S3 Particle sizes (nm) of the reconstructed LDH_{350} in CO_3^{2-} estimated by Sherrer's equation.

	0.2 eq	3 eq
0.5 h	8.02 nm	9.03 nm
4 h	9.03 nm	11.4 nm
12 h	12.9 nm	13.7 nm

2. AFM images

Fig. S3 showed the 3-dimensional AFM images and corresponding height profile of pristine LDH and LDO. As clearly seen, the surface of both LDH and LDO, regardless of lateral dimension, was very flat (smooth). The surface roughness value Ra of LDH_{2000}, LDO_{2000}, LDH_{350}, and LDO_{350} are 37, 35, 0.8 and 0.8 nm, respectively.

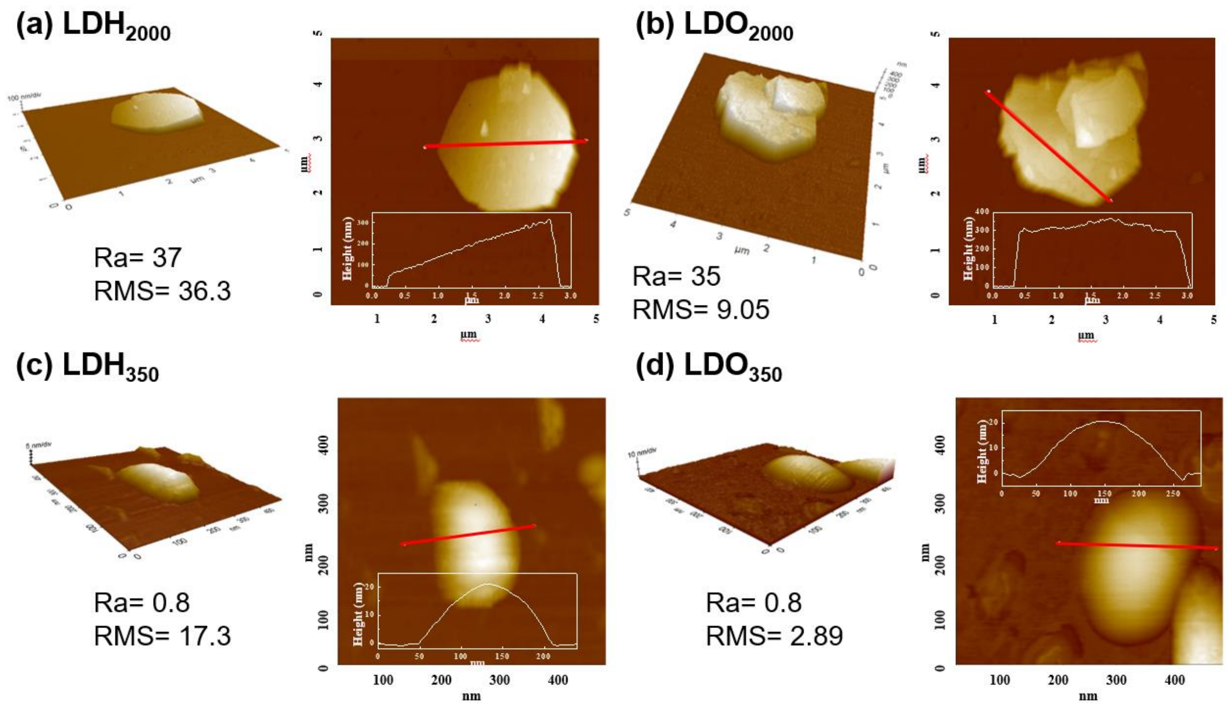

Fig. S1 Atomic force microscopic (AFM) images of (a) LDH_{2000}, (b) LDO_{2000}, (c) LDH_{350} and (d) LDO_{350}.

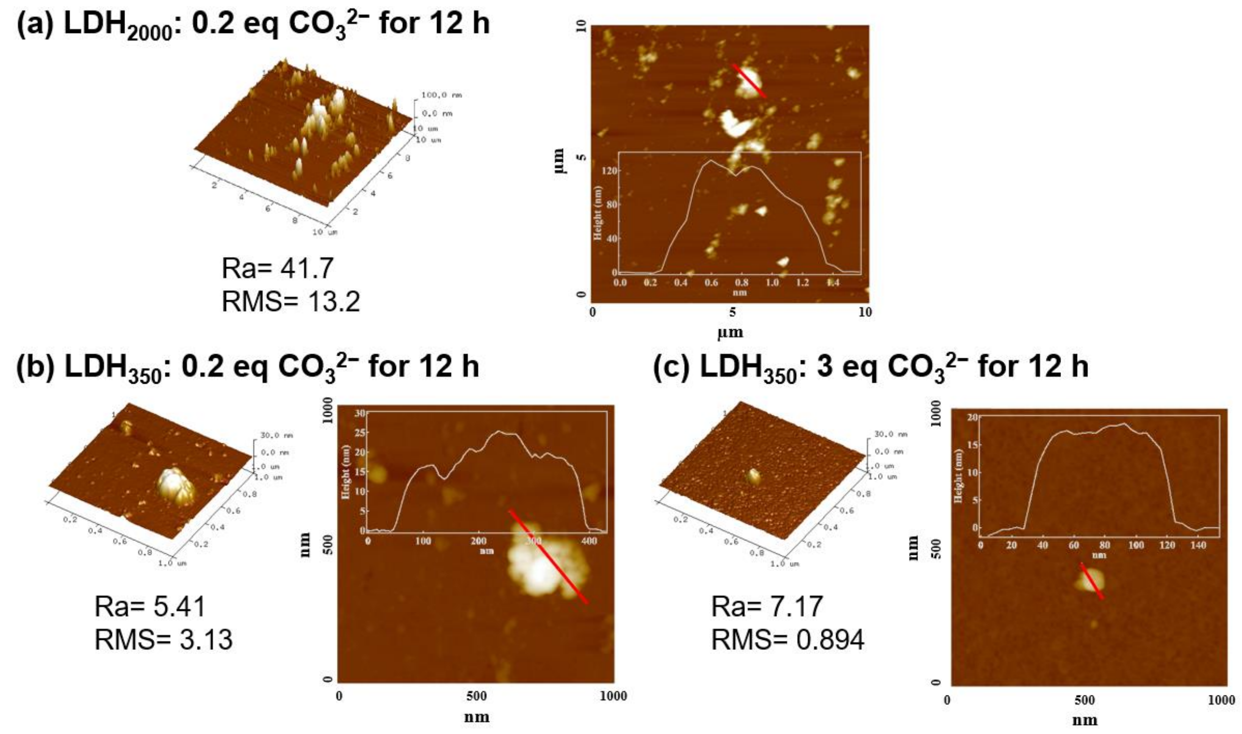

Fig. S2 Atomic force microscopic (AFM) images of the reconstructed LDH_{2000} and LDH_{350} by stirring in the aqueous $NaHCO_3$ solutions at 0.2 eq and 3 eq for 0.5 h and 12 h.

Fig. S3 and S4indicated the AFM images and height profile of LDH reconstructed with MO at representative concentration and time point. As we could clearly observe in the profile, the surface of reconstructed LDH was rougher than pristine; the surface roughness becomes more significant with increasing MO concentration in eq unit (which is also strongly related to loading ratio) as well as reaction time.

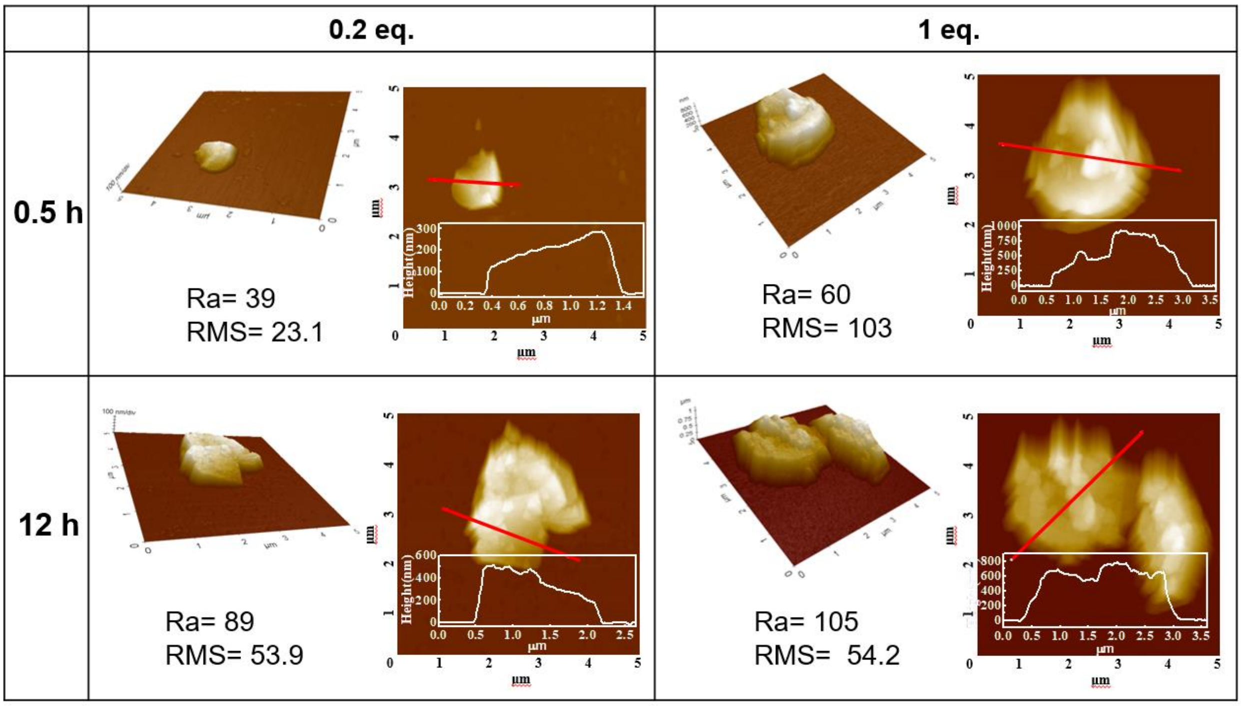

Fig. S3 Atomic force microscopic (AFM) images of the reconstructed LDH_{2000} by stirring in the aqueous MO solutions at 0.2 and 1 eq for 0.5 and 12 h.

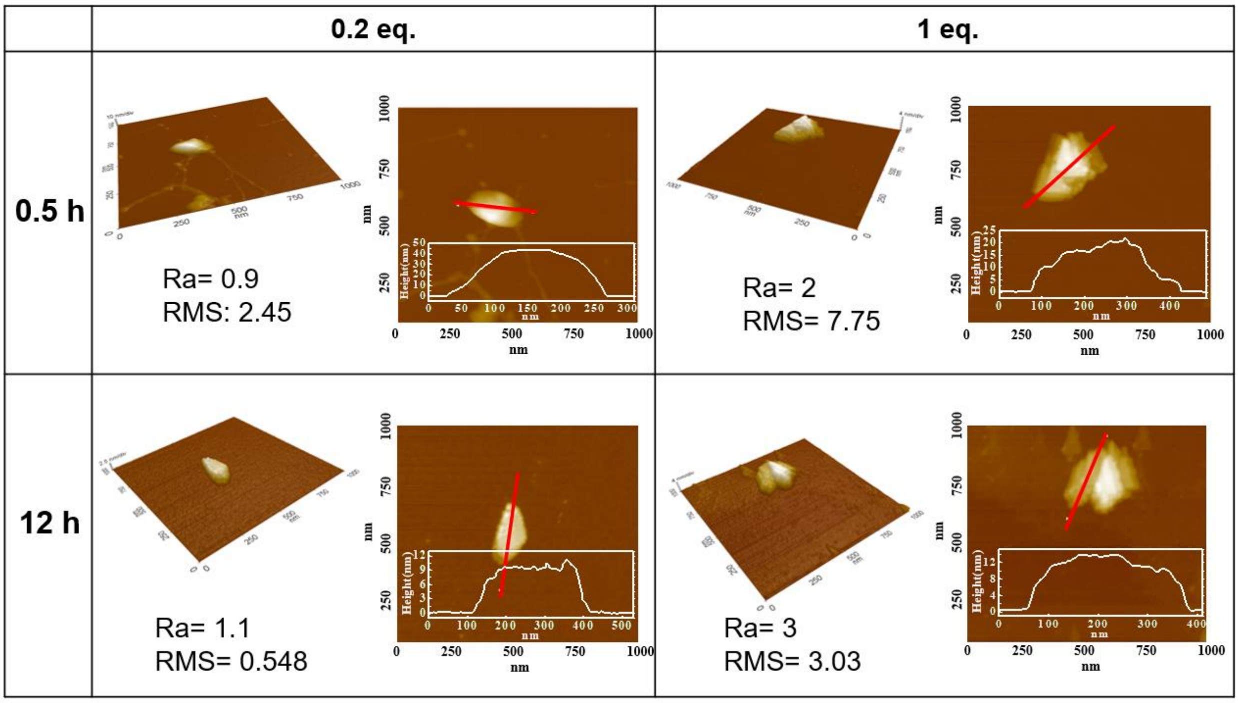

Fig. S4 Atomic force microscopic (AFM) images of the reconstructed LDH_{350} by stirring in the aqueous MO solutions at 0.2 and 1 eq for 0.5 and 12 h.
